# Landmark studies and emerging strategies for the management of acute severe ulcerative colitis

**Published:** 2019

**Authors:** Simcha Weissman, Saad Saleem, David Aldulaimi

**Affiliations:** 1 *Department of Medicine, Touro College of Osteopathic Medicine, Middletown, NY, USA*; 2 *Department of Medicine, Mercy Saint Vincent Medical Center, Toledo, OH, USA*; 3 *Department of Gastroenterology, South Warwickshire Foundation Trust, Warwick, UK*

Des Inflammatory bowel disease (IBD) is a systemic, inflammatory, autoimmune disorder marked by periods of disease flares and clinical remission. The two main components of IBD are Crohn’s disease (CD) and Ulcerative colitis (UC). Although its etiology is unknown, IBD is believed to be due to an exaggerated inappropriate immune response to luminal antigens leading to significant systemic inflammation. Although IBD was historically a disease with a high mortality, therapeutic advances have led to greatly improved patient outcomes. Despite these advances, it is estimated that 25% of patients with UC will require hospitalization for acute disease at some point along their disease course. Acute UC is a severe, life-threating situation that acquires immediate medical attention. Corticosteroids are the cornerstone of management and highly efficacious; however, approximately a third of patients will fail steroid therapy. As such, many new agents were developed, and some are still undergoing trials for consideration to be among the armamentarium for acute severe UC. Moreover, there has been renewed interest in lifestyle risk factors for UC.

In this editorial we review the landmark studies and some of the emerging treatment strategies, both medical and preventative, in the management of acute severe UC. 

**Landmark studies**

Cyclosporine acts by directly inhibiting calcineurin—an important component of cytokine gene transcription. This terminates T-lymphocyte activity by down regulating interleukin (IL)-2, IL-3, IL-4, tumor necrosis factor (TNF)-alpha, granulocyte-macrophage colony-stimulating factor, and interferon-gamma. In a landmark study by Lichtiger *et al.* in an attempt to determine the efficacy of cyclosporine, cyclosporine or a placebo was administered to 20 patients with steroid refractory disease. The authors reported that 82% of the eleven patients responded rapidly to the intravenous (IV) cyclosporine (1). In a slightly larger, Moskovitz *et al.* reported that 83% of the 142 patients, not only responded to cyclosporine, but also avoided colectomy during hospitalization (2). A major limiting factor that exists with cyclosporine usage is the potential for nephrotoxicity in the acute setting calling for the continuous monitoring of blood pressure and kidney function. Hence, if serum creatinine rises greater than 25%, therapy should be terminated. In addition, seizures, paresthesia, hypertension, hyperkalemia, and gingival swelling were found to be prevalent (3). As a potent metabolite of the cytochrome P450 3A pathway, it has a delicate balance with regard to drug kinetics. P450 inducers such as phenytoin, carbamazepine, and octreotide reduce cyclosporine blood levels, while inhibitors like erythromycin and ketoconazole increase cyclosporine levels.

Tacrolimus, also an inhibitor of calcineurin, acts by binding to the FK506 binding protein. Ultimately, as with cyclosporine, this leads to a decreased production of IL-2 and thus T-lymphocytes. In a multi-center study by Ogata *et al.* in an attempt to determine the efficacy of oral tacrolimus, they reported 50% of 63 patients with acute moderate-severe UC, had responded clinically to the oral medication. Additionally, 44% had significant mucosal healing only a few weeks in (4). In another study by Baumgart *et al.* the authors reported that 78% of 40 steroid dependent or steroid refractory patients with UC went into remission (5). Interestingly, diabetes, neurotoxicity, and nephrotoxicity are more common in tacrolimus than in cyclosporine recipients (6).

Infliximab is a chimeric monoclonal antibody with a potent biologic activity. Its mechanism of action calls for an inhibition of TNF-alpha and IL-1. This reduces downstream cytokine signaling, and therefore decreases systemic inflammation. In the monumental ACT 1 and 2 trials by Rutgeerts *et.al.* containing 364 UC patients each, the authors reported that infliximab had a higher clinical response at all time points. At week 8 over 30% of patients were in remission compared to 6% in the placebo group. In fact, sustained remission was achieved in 20% of patients compared to 5% in the placebo group. Additionally, mucosal healing was demonstrated in the infliximab group as early as week 8 (7). In a trial by Jarnerot *et al.* 45 patients with acute severe UC were administered infliximab purely as rescue therapy. Interestingly, the authors reported that infliximab had in fact reduced colectomy rates by double that of the placebo group (8). The adverse effects include mild injection sight reactions and infection while rare cases of hepato-splenic lymphoma have been reported (9).

In order to determine superiority in efficacy of cyclosporine versus infliximab, Laharie *et al.* performed an open label, randomized trial. Cyclosporine or infliximab was administered to 115 patients with severe, steroid refractory UC. They reported that at approximately, only in one week, 85% of patients in both groups responded clinically to treatment. Colectomy rates were also similar between the cyclosporine and infliximab groups. In conclusion, there was no clear evidence of superiority of any one of these regimens (10). A larger study by Willams *et al.* as well, reported no clear advantage to either therapy in terms of efficacy (11). This was then confirmed by a more recent study by Narula *et al.* In their meta-analysis of 16 different studies with over 1000 participants, no difference in efficacy was reported in either of the two-rescue therapy groups (12).

**Lifestyle hypothesis**

It has been accepted that genetic factors play a key role in the development of UC. Recently there has been renewed interest in the role of diet and exercise. It has been suggested that lifestyle changes can influence the guts microbiome, potentially modulating local and systemic immune activity.

**Studies looking at diet and exercise**

Harig *et al.* have published research suggesting that eating dietary fibers encourage certain species of colonic bacteria to produce short chain fatty acids. These short chain fatty acids—namely butyrate, acetate, and propionate—have been shown to suppress the colonic inflammation, especially in patients with UC (13). In an article by Jowett *et al.* the authors concluded that certain foods actually increased the likelihood of relapse for UC patients. The foods identified were red meat and alcoholic beverages (14). Rangan *et al*. in a further attempt to develop a connection between dietary intervention and IBD, tested a short fasting diet on dextran sodium sulfate a mouse model of IBD. The short fasting diet reduced intestinal inflammation, stimulated anti-inflammatory markers, and reversed the pathology caused by the dextran sodium sulfate (15). These studies suggest that dietary modification can modulate immune function in the colon and influence the likelihood of a clinical relapse.

In a study by Monda *et al.* the authors analysed the effects exercise had on gut flora in mice. The study revealed a correlation between physical activity level and microbial diversity. Furthermore, the authors reported increased colonic butyrate in those mice that underwent exercise as a result of colonization with colonic bacterial species (16). Recently, Stavsky *et al.* created a theoretical mechanistic framework on how diet and aerobic exercise have a synergistic role in the prevention and pathogenesis of UC. They suggest that with the increased intake of fibrous foods, reduction in intake pro-inflammatory amino-acids and physical activity may increase the production of short chain fatty acids that can modulate the immune response, leading to reduced colonic inflammation (17). 

**New medications and treatments**

Given the large number of patients whom fail steroid therapy and/or are not fortunate enough to be salvaged by infliximab or cyclosporine as rescue therapy, there is a substantial need for future medications to aid in the prevention of colectomy for acute severe UC. Tofacitinib is an orally administered, small molecule that blocks Janus kinase (JAK) 1,2 and 3. The inhibition of JAK—a signal transducer and activator of gene transcription—allows for a decreased production of numerous pro-inflammatory cytokines. After speedily progressing through numerous trials, tofacitinib was recently approved by the Food and drug administration (FDA) for UC. In the first trial to extend its use to patients with acute severe UC, Berinstein *et al. *tested its efficacy in four patients. Out of the four patients they reported, only one was unable to achieve clinical remission. Moreover, 50% of them rapidly responded to the oral therapy and completely avoided colectomy (18). In a larger study by Hanuer *et al.* post-hoc analysis of data from two-phase III trials demonstrated its efficacy over the placebo groups in inducing remission in a three-day period (19). In regards to its adverse effects, only infectious disease phenomenon have been reported similar to that of the biologics. Although tofacitinib seems very promising, more studies need to be done as to its efficacy in the acute severe setting. 

Anakinra is a potent IL-1 blocker with potential to inhibit neutrophil recruitment and thus prevent the early stages of inflammation. Its rapid action, short half-life, and broad inhibition of multiple IL-1 subtypes, make it a promising therapy. The trial by Thomas *et al.* dubbed—Interleukin 1 blockade in acute severe colitis (IASO)—is currently testing whether anakinra, given with corticosteroids, can reduce the need for rescue therapy and/or surgery in patients with acute severe UC (20). In addition, its safety profile is being expanded and is expected to complete its phase II trial in the near future.

Hyperbaric oxygen is an alternative method in an attempt to avoid rescue therapy and/or surgery. It is postulated that the pure excess oxygen negates the tissue hypoxia caused by UC. In a study by Jairath *et al.* hospitalized patients with acute severe UC were reported to show great efficacy to short term therapy with hyperbaric oxygen (21). In a few larger studies, hyperbaric oxygen in conjugation with corticosteroids achieved clinical remission and even prevented an escalation of medical therapy in numerous patients (22). Studies also reported its safety and efficacy in preventing surgery (23). Despite promising results being reported in studies, hyperbaric oxygen is yet to gain widespread use, probably reflecting limited access to this treatment. 

In conclusion, while acute severe UC is a serious medical condition requiring immediate multi-disciplinary action, patient outcome has been greatly improved. Since the advent of corticosteroids, the emergence of cyclosporine and infliximab have attributed immensely to this phenomenon. However, the reality is that some patients do not respond to either therapy, and therefore require colectomy. With the continued development of small molecules, alternative therapies and lifestyle recommendations, new treatment options for acute severe UC patients continue to evolve. More studies need to be done to determine an effective algorithm to better strategically position all these new agents in the armamentarium for acute, steroid refractory and severe UC.
